# Anesthetized animal experiments for neuroscience research

**DOI:** 10.3389/fncir.2024.1426689

**Published:** 2024-05-30

**Authors:** Shin Nagayama, Sanae Hasegawa-Ishii, Shu Kikuta

**Affiliations:** ^1^Department of Neurobiology and Anatomy, McGovern Medical School at the University of Texas Health Science Center at Houston, Houston, TX, United States; ^2^Pathology Research Team, Faculty of Health Sciences, Kyorin University, Mitaka, Japan; ^3^Department of Otorhinolaryngology, Medical School of Nihon University, Tokyo, Japan

**Keywords:** anesthesia, brain, experimental animal, anesthetic-activated cells, sensory representation

## Abstract

Brain research has progressed with anesthetized animal experiments for a long time. Recent progress in research techniques allows us to measure neuronal activity in awake animals combined with behavioral tasks. The trends became more prominent in the last decade. This new research style triggers the paradigm shift in the research of brain science, and new insights into brain function have been revealed. It is reasonable to consider that awake animal experiments are more ideal for understanding naturalistic brain function than anesthetized ones. However, the anesthetized animal experiment still has advantages in some experiments. To take advantage of the anesthetized animal experiments, it is important to understand the mechanism of anesthesia and carefully handle the obtained data. In this minireview, we will shortly summarize the molecular mechanism of anesthesia in animal experiments, a recent understanding of the neuronal activities in a sensory system in the anesthetized animal brain, and consider the advantages and disadvantages of the anesthetized and awake animal experiments. This discussion will help us to use both research conditions in the proper manner.

## Introduction

Anesthesia could be reversibly induced by the anesthetic agents and makes the brain and body condition into the following specific behavioral and physiological traits (Brown et al., 2010). 1. Analgesia: Animals do not perceive pain. 2. Unconsciousness: Animals are not aware of what’s happening. 3. Amnesia: Animals do not form memories. 4. Akinesia: Animals cannot move. Anesthesia decreases the painful stress and is helpful for the operation of the surgery on humans and animals. In addition, anesthesia helps monitor body and brain activity to understand the function at molecular, cellular, and circuit/tissue levels *in vivo*.

Brain science took advantage of anesthesia and revealed brain functions in the past centuries. In the recent decade, multiple experimental tools for monitoring the body and brain conditions have succeeded in becoming more compact and attachable to the body, directly allowing us to decrease animal stress and monitor the brain or body activity while behaving animals in awake conditions. In addition, the experimental procedures also improved to decrease the animal stress. Then, it decreased the hurdles to understanding the animal in awake conditions. The current trend is to study brain function in awake animal conditions. However, anesthesia is an important step for animal surgery, which is also necessary for many awake animal experiments, and it still has advantages in conducting experiments to understand brain function. In this review, we will shortly summarize the molecular mechanisms of popular anesthesia and clarify the difference between awake and anesthetized animal experiments to have the appropriate experimental conditions for the anesthetized and awake animal experiments.

### How do anesthetic agents work?

Anesthetic agents affect the animal brains and modify regular neuronal activity (Franks and Lieb, 1994). Although we frequently use anesthesia for animal experiments, we still do not understand the molecular, cellular, and circuitry mechanisms of how anesthetic agents induce Analgesic, Unconsciousness, Amnesic, and Akinetic conditions (Århem et al., 2003). Here, we quickly summarize the current knowledge of the molecular mechanism of the following major anesthesia (Pentobarbital, Ketamine, Urethane, and Isoflurane), in addition to the major sedatives (Xylazine, Chlorprothixene, and Dexmedetomidine) (Table 1).

**Table 1:** Anesthetic agents and sedatives.

Name	Target receptors/channels	Effects and features	References
Pentobarbital	GABA_A_ receptor agonist	Impacts on the medullary system Depresses cardiovascular and respiratory activities	Field et al. (1993), Franks (2008), Dutton et al. (2019), Johnson and Sadiq (2022)
Ketamine	NMDA receptor antagonist	Increase heart rate, blood pressure, and cardiac output	Kurdi et al. (2014), Stoicea et al. (2016)
Urethane	Potentiate nicotinic acetylcholine, GABA, glycine receptors Inhibit NMDA, AMPA receptors	Minimal effects on cardiovascular and respiratory systems, and maintenance of spinal reflexes Spontaneous brain state alterations similar to natural sleep Carcinogen	Maggi et al. (1986), Hara and Harris (2002), Pagliardini et al. (2013)
Isoflurane	Allosteric modulator of GABA_A_ Enhance the activity of glycine receptor, NMDA receptor agonist Inhibit the potassium channel conductance	Volatile anesthesia Increases and decreases the extracellular potassium and sodium concentration by the Na^+^/K^+^ -ATPase impairment, respectively	Grasshoff and Antkowiak (2006), Dickinson et al. (2007), Reiffurth et al. (2023)
Xylazine	α_2_-adrenergic receptor agonist	Often used together with Ketamine Anesthesia, muscle relaxation, analgesia, hypotension, respiratory depression	Ruiz-Colón et al. (2014)
Chlorprothixene	Serotonin (5-HT_2_), Dopamine (D_1_, D_2_, D_3_), Histamine (H_1_), Muscarinic, and α_1_-adrenergic receptors antagonist	Often used together with Urethane or Ketamine/Xylazine	Højlund et al. (2022)
Dexmedetomidine	α_2_-adrenergic receptor agonist	Inhibits the norepinephrine release from locus coeruleus Low respiratory depression	Gertler et al. (2001), Franks (2008)

Detailed features of the anesthetic agents were summarized in previous works (Sakai et al., 2005; Sorrenti et al., 2021).

Pentobarbital: Pentobarbital have been one of the major anesthetic agents. It binds to the gamma-aminobutyric acid (GABA) type A (GABA_A_) receptors (Franks, 2008; Johnson and Sadiq, 2022). It induces chloride channels to open longer, potentiate GABA effects, and induce longer hyperpolarization. The advantage of Pentobarbital anesthesia would be its reliability to induce rapid unconsciousness. It impacts the sensory systems and the medullary and depresses cardiovascular and respiratory activities (Field et al., 1993; Dutton et al., 2019). It also has the function of sedation.

Ketamine: Ketamine targets *N*-methyl-d-aspartate (NMDA) receptors to reduce the excitatory action of glutamate (Stoicea et al., 2016). Its prominent features as anesthesia are increased heart rate, blood pressure, and cardiac output, mediated principally through the sympathetic nervous system (Kurdi et al., 2014). It has small effects on the central respiratory drive. It increases salivation and muscle tone. Ketamine also has the function of an antidepressant.

Urethane: Urethane is one of the most popular anesthetics used for animal experiments for a long time. Although it is a carcinogen and is not proper for survival surgery, it is still well used because of its minimal effects on cardiovascular and respiratory systems, and it could maintain spinal reflexes and spontaneous brain state alterations similar to natural sleep (Maggi et al., 1986; Pagliardini et al., 2013). In addition, it can produce a long-lasting steady level of anesthesia during surgery and experiments. It is assumed that animals anesthetized with urethane represent similar physiologic and pharmacologic behaviors to those observed in unanesthetized animals. However, little is known about its mechanism. Recent research revealed the urethane effect of multiple ion channels (Hara and Harris, 2002). Urethane potentiates the functions of nicotinic acetylcholine, GABA, and glycine receptors, and it inhibits NMDA and α-amino-3-hydroxy-5-methyl-4-isoxazole propionic acid (AMPA) receptors in a concentration-dependent manner. Because urethane had modest effects on all channels tested in the experiment, urethane would not have a single predominant target molecule for anesthesia.

Isoflurane: Isoflurane is volatile anesthesia. Therefore, one of the prominent features of isoflurane anesthesia is the easier control and faster anesthesia induction and recovery. The molecular mechanism of isoflurane anesthesia has not been well known (Jones et al., 1992; Jenkins et al., 1999; Krasowski and Harrison, 2000). Isoflurane acts as an allosteric modulator of the GABA_A_ receptor, enhances the activity of glycine receptors, and decreases motor function (Grasshoff and Antkowiak, 2006). Isoflurane inhibits the activity of NMDA receptors at the same site as glycine (Dickinson et al., 2007). Isoflurane inhibits the conduction of the potassium channel (Buljubasic et al., 1992). In addition, Isoflurane also works as a burst suppression anesthesia, and increases the extracellular potassium concentration and decreases extracellular sodium concentration by the Na^+^/K^+^-ATPase impairment (Reiffurth et al., 2023).

These anesthetic agents are often used with Sedatives. The following three sedatives are popular in animal experiments.

Xylazine: Xylazine is often used together with Ketamine as a sedative, and the mixture of them is referred to as Ketamine/Xylazine (K/X). Xylazine also has effects on anesthesia, muscle relaxation, and analgesia. Xylazine is known as an α_2_-adrenergic receptor agonist (Ruiz-Colón et al., 2014). It also has side effects of hypotension and respiratory depression.

Chlorprothixene: Chlorprothixene is often used together with Urethane or Ketamine/Xylazine. It is an antipsychotic drug (Højlund et al., 2022). Chlorprothixene has strong impacts by blocking the Serotonin (5-HT_2_), Dopamine receptors (D_1_, D_2_, D_3_), Histamine (H_1_), Muscarinic, and α_1_-adrenergic receptors.

Dexmedetomidine: Dexmedetomidine induces sedation by agonistically binding to α_2_-adrenergic receptors and inhibits the norepinephrine release from locus coeruleus in the brain stem (Gertler et al., 2001; Franks, 2008). Unlike opioids and other sedatives such as propofol, dexmedetomidine can achieve its effects without causing respiratory depression.

This summary shows us that different anesthetic agents and sedatives target different ion channels or transmitter receptors. However, they induce a similar trait of anesthesia.

### Neuronal mechanism of sleep and anesthesia

General anesthesia and natural sleep induce similar behaviors. Therefore, their similarities have been discussed for a long time (Shafer, 1995; Date et al., 2020). The two conditions have both similar and different functional features. Recent research revealed that sleep is a more active process than previously considered. Many of the reports especially focus on the hypothalamus area (Szymusiak et al., 2007; Sternson, 2013; Wu et al., 2014; Scott et al., 2015; Tan et al., 2016; Allen et al., 2017). It is reported that some given groups of neurons in the hypothalamus show activity under sleep or anesthesia conditions (Moore et al., 2012; Zhang et al., 2015; Gelegen et al., 2018). These neurons are called anesthetic-activated cells. More recent research revealed that anesthetic-activated cells contribute to inducing and maintaining the anesthetized and sleep conditions, in addition to the awake condition.

Jiang-Xie’s group found the anesthesia-activated cells in the supraoptic nucleus in the hypothalamus. An interesting finding was that if they activate the neuron’s chemogenetic or optogenetic method, the animal stops moving and falls into a slow wave sleep condition. If the neurons are conditionally ablated or inhibited, the mice continuously move around and cannot fall asleep. In addition, if they silence the activity of the neurons, the mice work up from anesthesia easily (Jiang-Xie et al., 2019).

Opposite functional types of neurons, whose activity is associated with induction and maintenance of awake condition, were also found. Reitz’s group showed that chemogenetic activation of tachykinin 1 expressing neurons in the preoptic area obliterates both non-rapid eye movement (NREM) and rapid eye movement (REM) sleep. Moreover, chemogenetic activation of these neurons stabilizes the waking state against both Isoflurane- and Sevoflurane-induced unconsciousness (Reitz et al., 2021).

These researches shed light on that the specific neurons and neuronal circuits in the hypothalamus would contribute to induce and maintain the anesthetized and sleep conditions, in addition to awake conditions. The anesthetic agents described in the previous section may have a strong effect on these specific neurons in the hypothalamus. However, the anesthetic agents also impact other neurons, which may not be directly associated with the induction and maintenance of sleep, anesthetized, and awake animal conditions. Further study of the molecular cellular and circuitry contributions to the induction and maintenance of sleep, anesthetized, as well as awake animal conditions, are demanded. The knowledge will help to interpret the functional research data collected in anesthetized animal conditions correctly and help to improve the method of anesthesia beyond the current anesthetic agents and procedures.

### Sensory response in anesthetized and awake condition

Anesthesia induces Analgesia, Unconsciousness, Amnesia, Akinesia in animals. Therefore, the downstream (or outputs) of the neuronal circuits associated with these behavioral and psychological traits would be strongly disturbed during anesthesia. Indeed, a large number of the research reported the differences in activity of neurons, neural population, and functional connectivity of brain areas between anesthetized and awake conditions (Nallasamy and Tsao, 2011; Sellers et al., 2015; Hu et al., 2024). However, the upstream and peripheral of each circuit, such as sensory representation in the primary or secondary sensory areas, may be less disturbed by anesthesia.

In the primary visual cortex, anesthetics reduce spontaneous neuronal spike activity but promote synchronized activity (Greenberg et al., 2008; Aasebø et al., 2017; Lee et al., 2021). The change is larger in Isoflurane compared to the Ketamine/Xylazine anesthesia (Aasebø et al., 2017). Interestingly, the narrow spiking neurons (presumably inhibitory neurons) have a larger decrease in spontaneous spike activity compared to the broad spiking neurons (presumably excitatory neurons) in anesthesia (Aasebø et al., 2017). To the visual stimulation, anesthetized animals showed prolonged neuronal activity by the stimulation in a wide area in visual space. In contrast, neuronal responses to visual stimulation are more spatially selective and much briefer during wakefulness. The difference is owed to the strong inhibition of extremely broad spatial selectivity during wakefulness (Haider et al., 2012).

In the olfactory bulb, wakefulness greatly enhances the activity of inhibitory interneurons of granule cells. As a result, the odor responses of the principal neurons of mitral cells are not prominent compared to those under anesthetized animals. However, awake animals show more sparse and temporally dynamic responses (Kato et al., 2012; Blauvelt et al., 2013; Wachowiak et al., 2013). In addition, repetitive odor experiences in awake animal condition weaken the odor response of mitral cells gradually for a long time. This mitral cell plasticity is odor-specific, recovers gradually over months, and can be repeated with different odors. Furthermore, the expression of this experience-dependent plasticity is prevented by anesthesia (Kato et al., 2012).

In the barrel cortex, electrophysiological responses evoked by whisker deflection are reduced in amplitude under anesthetized condition (Simons et al., 1992; Devonshire et al., 2010). However, delayed activity, probably due to the inputs from the neighboring whisker stimulation, is more prominent and prolonged. The reduction in response amplitude was considered by the global down-scaling of the population response. Interestingly, the variation of the spike frequency is larger during wakefulness. The difference is more prominent in layer 5A, especially during whisking episodes (De Kock and Sakmann, 2009).

These researches indicate that sensory representation in the primary sensory areas could be observed in both anesthetized and awake animals. However, the amplitude, tuning specificity of sensory inputs, and temporal pattern of neuronal activity are not completely the same in anesthetized and awake animal conditions. Importantly, the anesthesia delivers different impacts on the different layers, neuronal types, and sensory systems. The majority of the reasons for the difference have not yet been clearly determined. However, it probably owes to the combination change of the direct or indirect pharmacological impact of anesthetic agents on the recording neurons and the top-down signals to each recording neuron between the anesthetized and awake animal conditions.

## Advantages and disadvantages of anesthetized animal research

Advantages: Anesthetized condition is a pharmacologically induced artificial condition, which is useful for operating surgery and physiological experiments. The akinetic effect minimizes animal movement and helps us obtain high-quality experimental data such as *in vivo* electrophysiology or functional optical imaging experiments. In addition, the analgesic effect decreases the pain sensation and helps to decrease the animal stress of pain or uncomfortable procedures of surgery or experiments. Therefore, the biggest advantage of the anesthetized animal experiment would be that it allows us to conduct functional experiments easily and reliably.

Disadvantage 1: One of the problems of the anesthetized animal experiment is the broad and unidentified impacts of anesthetic agents on the brains. The normal neuronal activity and response to the neuronal transmitters are disturbed by the anesthetic agents. Probably, some synaptic transmission and neuronal activity would be enhanced, and others would be inhibited to some degree. In other words, the brain activity is modified in an anesthetic agent specific manner in anesthetized animals. These impacts are more remarkable when we monitor the neuronal activity in higher brain centers, where the sensory inputs reach after many synaptic transmissions. Therefore, functional research on the higher brain center may not be adequate under anesthesia in general. However, some of the peripheral sensory brain areas, which reached the sensory inputs after the small number of synaptic transmissions, would be less impacted by the anesthesia, although the centrifugal or top-down signal would be abnormal or less active in this case.

Disadvantage 2: Sensory information is actively acquired in awake-behaving rodents, such as active movement of whiskers and an increase in the sniffing cycle in olfaction (Petersen, 2007; Wachowiak, 2011). Anesthetized animals do not have such behavioral outputs. Therefore, the research using anesthetized animals allows us only to study the passive sensory processing without animal behavior attempting to intensely collect and recognize the sensory inputs. It would make neuronal response simpler and easier to interpret. However, we need to carefully interpret the data concerning the lack of active sensing top-down signals, which contribute to sensory recognition in the process of perception.

## Advantages and disadvantages of awake animal research

Advantage: There is no doubt that animal experiments in awake conditions are one of the ideal functional experimental conditions because they allow us the naturalistic neuronal activity in the experiment. In addition, some of the disadvantages of the anesthetized animal experiments discussed above are fully or partially overcome by the awake animal experiments.

Disadvantage 1: One of the weak points of the awake animal experiments is that we could easily capture the large size of noise associated with the animal movement. Recent progress in experimental skills, such as solid attachment of the recording systems on the skull, remarkably improved the weakness. In addition, researchers were intensely challenged to compensate for the movement artifacts during the offline data analysis. As a result, we can now collect more reliable and high-quality neuronal activity in awake animals than we did previously. However, monitoring neuronal activity using recording methods sensitive to animal movement, such as *in vivo* intracellular or patch-clamp recording methods, is still challenging.

Disadvantage 2: Some of the experimental systems are too large and heavy to attach to the animal brain, such as optical imaging systems with high numerical aperture lenses. Some probes, such as functional ultrasound imaging systems, functional magnetic resonance imaging systems, etc., require to be moved while monitoring the brain functions. The head-restrained animal experiment would be the best choice for using such equipment. However, the head-restrained animal researches require a relatively short time experimental duration (~1 h) to decrease the head-restrained stress of animals. If it requires a longer (more than 6 h) duration, the anesthetized animal experiments have benefits. For example, the functional mapping of relatively peripheral areas, such as the olfactory bulb, barrel cortex, visual cortex, etc., has the advantage of using the anesthetized experiment.

Disadvantage 3: Massive volume of information is processed in awake animal brains simultaneously. It is one of the biggest benefits of the awake animal experiment, which allows us to understand the naturalistic brain function *in vivo*. However, it also has a tradeoff. Too much activity makes it difficult to capture or extract the critical signals among the flood of massive neuronal activities. Turning off some of the neuronal activity or neuronal pathways in awake animal experiments may help to dissect the brain function and focus on discussing the specific neuronal activity and pathways in some cases.

Disadvantage 4: Usually, the recording system is attached over the skull, or the recording probes are implanted in the target brain area under anesthesia before the awake animal experiment. In this case, we need to consider postoperative delirium to have the proper recovery time after the anesthetized surgery (Peng et al., 2016). Even if the animal shows normal activity after the surgery, animal performance for some experimental tasks may not be the same as that before the surgery. Therefore, the careful evaluation of the animal recovery from the anesthetized surgery is required for the awake animal experiments associated with anesthetized animal surgery.

## Discussion

Considering the advantages and disadvantages discussed above session, the current method of anesthesia would be advantageous in some of the experiments, such as (1) Mapping the passive sensory inputs, (2) Evaluating the synaptic interaction or pharmacological impact in the local circuit, and (3) Measuring the neuronal activity under stressful in awake animal conditions.

In addition, some of the disadvantages of awake animal experiments will be overcome by the progress of technologies in the future. For example, the large and heavy recording systems will become more compact and lighter. They will be less stressed when attached to the animal skull. Therefore, these disadvantages could be partially solved in the future.

Recent studies showed the potential that anesthesia and sleep conditions may be inducible by the control of the specific neuronal types and circuits. These discoveries will shed light on the potential of new types of anesthesia. Suppose we will be able to control the activity of the proper number and group of anesthetic/awake associate neurons by chemogenetic or optogenetic tools and control the anesthetized/awake states of animals in the future. In that case, this procedure will become a new type of anesthesia and minimize the impact of anesthesia on other brain functions such as respiration and cardiovascular systems. It will increase the controllability of the anesthesia, may decrease the animal death associated with the failure control of the respiratory and cardiovascular systems, and increase the success rate of the animal surgery. In addition, if we know the specific neurons and neuronal circuits affected by the anesthesia, we could avoid studying these neurons and circuits, and focus on studying the other neuronal circuits, which have normal activity of received minimum impact of the new anesthesia, as like awake animal research more easily and efficiently than current awake animal experiments. Understanding the essential neuronal and circuitry mechanisms that induce sleep/anesthesia and awake animal conditions would extend the anesthetized animal research beyond the current limitation and help to understand the more naturalistic brain function in anesthetized animal conditions.

## Summary

In this review, we attempt to summarize the molecular mechanism of popular anesthetic agents, the relationship between hypothalamic neurons and anesthesia/sleep/awake conditions, sensory activities under anesthesia, the advantages and disadvantages of anesthetized and awake animal experiments, and discuss the potential of future anesthesia. This would help in conducting anesthetized experiments and future animal research.

## Author contributions

SN: Writing – original draft, Writing – review & editing. SH-I: Writing – review & editing. SK: Writing – review & editing.
